# Golgipathies reveal the critical role of the sorting machinery in brain and skeletal development

**DOI:** 10.1038/s41467-022-35101-y

**Published:** 2022-12-01

**Authors:** Vincent El Ghouzzi, Gaelle Boncompain

**Affiliations:** 1 NeuroDiderot, Inserm, Université Paris Cité, F-75019 Paris, France; 2 Institut Curie, PSL Research University, CNRS UMR144, Paris, France

**Keywords:** Membrane trafficking, Diseases

## Abstract

Association genetic studies and genome-scale CRISPR screens have recently identified ARF3 and TMEM251/LYSET/GCAF as Golgi-resident factors essential to brain and skeletal development. Here we discuss how even though the consequences of mutations in these genes affect endosomal and lysosomal compartments, the problem originates in the Golgi complex and may involve either the identity of the carrier vesicles or that of cargo molecules.

Intracellular trafficking is a highly dynamic and finely regulated process that ensures development and homeostasis in all cell compartments. At the heart of these pathways, the Golgi complex (GC) plays a central role in the recognition of neosynthesized proteins and lipids to tag them post-translationally, sort and package them in appropriate carriers and address them to their destination of interest. A logical consequence is that primary dysfunctions of the GC lead to variable and heterogeneous pathologies affecting multiple organs. If confirmed by the increasing number of disease-related genes associated with the GC, brain and skeletal development appear strikingly often affected by primary impairments of the sorting machinery^1^. In a series of studies just published in Nature Communications^2,3^, Science^4,5^ and Human Mutation^6^, two new Golgipathies associated with the GTPase ARF3 and TMEM251 highlight this particular vulnerability of the nervous system and skeletal development and reveal that the destiny of both carriers and cargos is determined in the Golgi.

## ARF3, a second member of the ARF family implicated in a developmental disorder

ADP-Ribosylation factors (ARFs) are highly conserved GTPases involved in the formation of carrier vesicles. Through their ability to recruit coat proteins at the surface of budding vesicles and interact with various adaptor proteins (AP), ARFs contribute to specify membrane carriers and promote cargo transport. All human ARFs except ARF6 are localized to the GC and so far only ARF1 has been associated with human pathogenic variants, in patients with developmental delay and brain malformation^7,8^. ARF1 share 96% aminoacid identity with ARF3 and it is assumed that their functions are partially interchangeable throughout the GC although studies have shown that ARF3 preferentially localizes to the TGN and is more specifically involved in the endosomal recycling pathway while ARF1 functions to both the *cis* and *trans* sides of the GC^9,10^. Human causal genetic variants have now been identified in ARF3 as well, in patients with a developmental disease impairing brain and skeletal development^2,11^. Most individuals with severe illness display progressive microcephaly, intellectual delay, delayed myelination and epilepsy, as well as scoliosis and thoracic abnormalities, defects typically reported in other developmental Golgipathies^12,13^. Interestingly, most variants target the GDP/GTP binding pocket of ARF3 by which ARF Guanine Nucleotide Exchange Factors (ARFGEFs) and ARF GTPase Activating Proteins (ARFGAPs) control its activation state, and these de novo variants appear to induce either blockage or maintenance of ARF3 activation. Importantly, expression of the variants in zebrafish phenocopy the human phenotype, whereas downregulating endogenous expression of fish *arf* genes does not, indicating a dominant mechanism of action^2^. The expression of the variants disorganizes the structural morphology of the GC and the formation of COP-I vesicles at the *cis* face of the Golgi, but also impacts the post-Golgi function of ARF3 in cargo recycling through the endosomal compartments, judging by significant delays in transferrin recycling and accumulation of unrecycled transferrin in lysosomes. These data suggest that the role of GC in controlling vesicle formation is disrupted in many cells and will impact tissue homeostasis throughout the life of the individual with variable defects and heterogeneity in severity and organs affected but do not explain the specific involvement of the brain and the skeleton. In an effort to address this issue, Fasano et al. showed that ARF3 also plays a role in early developmental processes such as the control of the mitotic spindle orientation in neural progenitor division during neurogenesis and planar cell polarity (PCP) that governs the collective morphogenetic events which organize initial body axes^2^. This role of ARF3 in cell polarity during development echoes an emerging function of the GC in post‐Golgi transport of polarity determinants to the apical surface of apical radial glial cells, a process that controls the balance between maintenance and delamination of neural progenitors during neocortex development^14^.

**Fig. 1:**
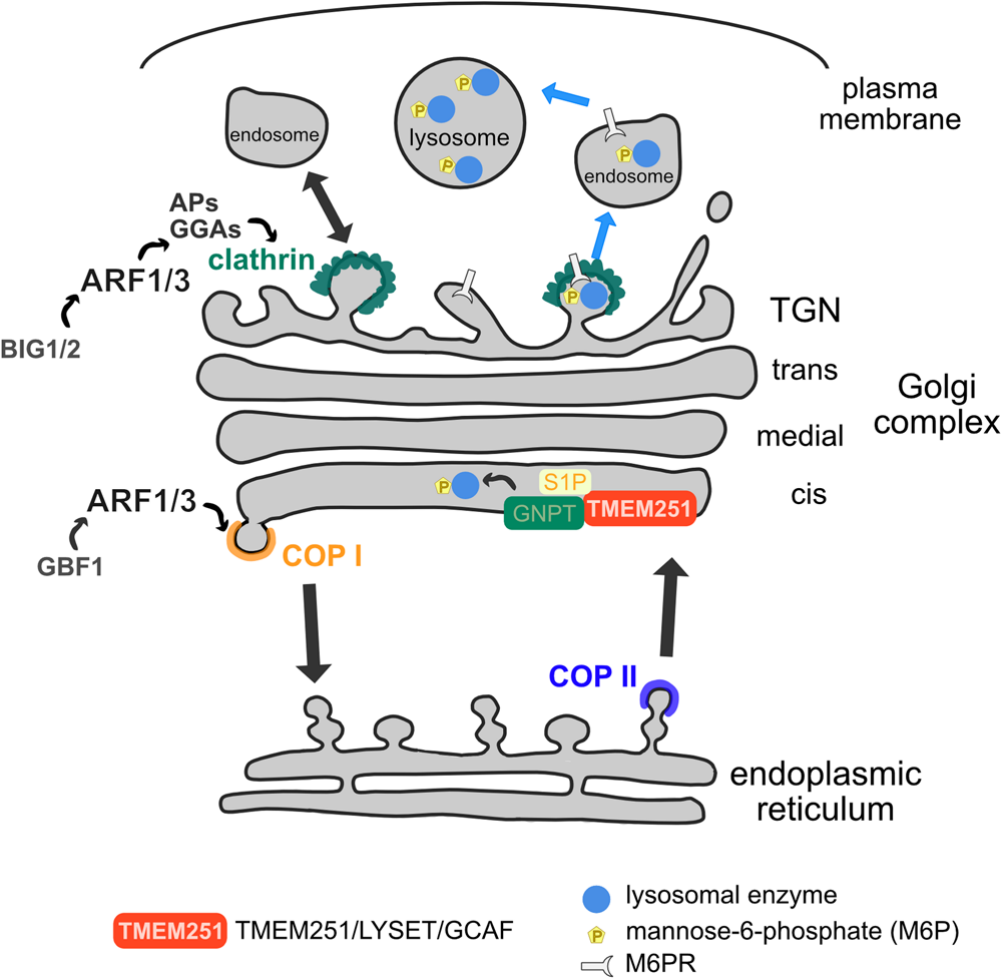
ARF1/3 are involved in the formation of carrier vesicles and LYSET is involved in the targeting of lysosomal enzymes. At the Golgi complex, ARF1/3 mediate the recruitment of coat proteins to give identity to the transport carriers. At the *cis*-Golgi and TGN, ARF1/3 recruit COP I and clathrin, respectively. TMEM251 is a Golgi protein required to add the mannose-6-phosphate (M6P) group on lysosomal enzymes, ensuring their proper addressing to the lysosome. Reprinted from “Transport and sorting in the Golgi complex: multiple mechanisms sort diverse cargo”, by G. Boncompain & A.V. Weigel, Current Opinion in Cell Biology 2018, 50:94–101, Copyright 2018, with permission from Elsevier Ltd.

Through the prism of ARF-regulated mechanisms, the control of membrane transport by the GC thus appears critical for both development and homeostasis of differentiated tissues. Of interest, the particular sensibility of the nervous and/or skeletal systems to transport vesicles can also be seen in patients with genetic variants in the ARFGEFs BIG1 and BIG2^15–17^, which promote ARF activation at the TGN, as well as in components of protein coats recruited by ARF proteins at both sides of the GC, such as COP-I coat proteins^18–21^ or cargo adaptors of the AP complex family involved in cargo sorting at the TGN^22–27^ (Table 1; Fig. 1). Again, the occurrence of both neonatal and progressive manifestations during childhood demonstrates the importance of the ARF pathway throughout development. Interestingly, although BIG1 and BIG2 may play redundant roles in trafficking between the TGN and endosomes^28–30^, the brain phenotype revealed by human mutations or deficient mouse models suggests that BIG1 controls maturation steps like peripheral myelination or synaptic transmission^30–32^ while BIG2 would rather play on the expansion and migration of neural progenitors^33^. Thus, one could speculate that ARF1/3 are differentially regulated at different stages of development, in progenitors or in post-mitotic cells, providing an explanation for the diversity and overlap of phenotypes observed. Ultimately, this demonstrates the developmental importance of mechanisms that regulate the transport of carrier vesicles between the GC and the cell surface.

**Table 1:** ARF1/3 partners and factors of the M6P pathway associated with human developmental diseases including brain and/or skeletal defects (y = yes; - = not reported)

Factor	Main function	Subcellular location	Brain phenotype	Skeletal phenotype	#MIM
ARF1	GTPase regulating transport vesicle formation	Golgi, TGN	y	–	103180
ARF3	GTPase regulating transport vesicle formation	Golgi, TGN	y	y	103190
BIG1	Guanine nucleotide Exchange factor (GEF) regulating ARF activation	TGN, endosomes	y	–	604141
BIG2	Guanine nucleotide Exchange factor (GEF) regulating ARF activation	TGN, endosomes	y	–	605371
COP-I	Coat protein complex of transport vesicles	Golgi, ER	y	–	600959 (COP1-B1)
			y	y	606990(COP1-B2)
			y	y	600820 (COP1-D)
AP1	Cargo adaptor	TGN, endosomes	y	y	603533 (AP1-G1)
			y	–	603531/300629 (AP1-S1/S2)
AP3	Cargo adaptor	TGN, endosomes, lysosomes	y	–	603401 (AP3-B1)
			y	y	607246 (AP3-D1)
AP4	Cargo adaptor	TGN, endosomes	y	–	607245/607244/ 602296/607243(AP4-B1/E1/M1/S1)
TMEM251	Docking GNPTα/β precursor in the Golgi	Golgi	y	y	619332
GNPT	Catalyzing the transfer of M6P on lysosomal enzymes	Golgi	y	y	607840/607838 (GNPTAB/GNPTG)
S1P	Proteolytic processing of the GNPTα/β precursor	Golgi	–	y	603355

## TMEM251, a new key piece in the mannose-6-phosphate pathway

Within the sorting machinery, the addition of the appropriate post-translational modifications is decisive for the correct targeting of proteins to their destination compartment. This is the case of the addition of the mannose-6-phospate (M6P) group on lysosomal enzymes to be targeted to the lysosome lumen. Again, the occurrence of Golgipathies tells us that this tagging mechanism takes place in the GC and its defective machinery leads to human diseases. A series of recent studies has highlighted the key role of TMEM251/LYSET/GCAF in the M6P pathway^3–5^. *TMEM251* is a gene encoding a *cis*-Golgi transmembrane protein in which mutations have recently been identified in patients with severe recessive skeletal dysplasia associated with metabolic abnormalities^6^. Patients display progressive severe symptoms throughout their lifespan. They show severe short stature and early mortality. Although very few patients have been described so far, a patient carrying an early stop mutation also showed brain abnormalities, including thinning of the corpus callosum. Abnormal activity of lysosomal enzymes has been detected in blood samples from patients. Altogether these symptoms were reminiscent of the lysosome storage disorder called mucolipidosis type II.

The elucidation of the function of TMEM251 and of its key role in the M6P pathway came from three concomitant CRISPR screens. Zhang et al. carried out a screen whose read-out was designed to detect proteins involved in lysosome function based on a dual fluorescent reporter fused to the fast degrading lysosome membrane protein RNF152^3^. Pechincha et al. conducted a screen to identify proteins involved in the generation of nutrient from extracellular proteins *via* lysosomal catabolism to sustain cell growth^4^. Richards et al. applied CRISPR screening to uncover proteins critical for reovirus infection, which requires lysosomal cathepsins activity to mediate disassembly of the viral particles^5^. The three studies demonstrated that knocking-out of TMEM251 led to many defects related to lysosomal functions. Increase in the autophagy marker LC3-II, in the number of lysosomes and the presence of undigested material in the lysosomes were detected. The key data conducting the three groups to in-depth investigation of the role of TMEM251 in the M6P pathway relied on the detection of the secretion of lysosomal enzymes in the extracellular medium.

Lysosomal enzymes are synthetized in the endoplasmic reticulum and are then transported to the GC. At the *cis*-Golgi, they undergo a series of enzymatic reactions leading to the addition of the M6P group. The first step is performed by GlcNAc-1-phopshotransferase (GNPT) and the second step by M6P-uncovering enzyme (UCE). GNPT is an hexamer which is assembled in the ER and requires cleavage by site-1-protease (S1P) that occurs at the *cis*-Golgi. Zhang et al. showed that TMEM251 KO phenocopies GNPTAB deficiency, abolishing M6P modification and indicating that TMEM251 acts upstream of GNPTAB. TMEM251 interacts both with GNPTAB and S1P. The three studies demonstrated that TMEM251 is required for the cleavage of GNPTAB. As in the ARF3 study, experiments performed in zebrafish, where TMEM251 is conserved, recapitulated skeletal defects observed in human patients. Either GNPTAB KO or TMEM251 KO generated severe edema and skeletal dysplasia^3^, and loss of cartilage and calcified structures was observed in mutant zebrafish.

Other players of the M6P pathway have been linked to developmental diseases in humans. Mucolipidosis type II and III are caused by a complete or partial loss of GNPT activity, respectively. Interestingly, symptoms also include brain and severe skeletal abnormalities^34,35^. Mutations in S1P has also been described to lead to skeletal defects^36^ (Table 1, Fig. 1).

## The sorting machinery at the heart of brain and skeletal development

The identification of ARF3 and TMEM251 mutations in genetic diseases and their involvement in the control of the sorting machinery underscore the critical role of the GC in the specific tagging of both carrier vesicles and cargo molecules. The congenital but also progressive nature of the symptoms illustrates that the dysfunctions generated affect the individual throughout life, affecting both key stages of fetal development and homeostasis of differentiated tissues. Why these Golgipathies particularly target the development of the skeleton and/or that of the brain still remains a mystery, although one can assume that cells which no longer divide or which require a particularly abundant secretion activity are likely more vulnerable. A limited regenerative capacity of cardiac cells could also explain the occasional involvement of the heart, known to be affected in lysosomal storage diseases, and whose defects have been observed in one of the TMEM251 patients^6^ and in the mutant zebrafish^3^. Alternatively, mechanisms specific to particular steps of neurogenesis and chondrogenesis could provide an explanation. The identification of other Golgipathies will no doubt continue to contribute to the understanding of the key functions of the GC during human development.
